# 肺腺癌所致乳糜胸1例

**DOI:** 10.3779/j.issn.1009-3419.2013.03.10

**Published:** 2013-03-20

**Authors:** 倩 王, 津娜 李, 殿胜 钟

**Affiliations:** 1 300052 天津，天津医科大学总医院肿瘤科 Department of Medical Oncology, Tianjin Medical University General Hospital, Tianjin 300052, China; 2 300052 天津，天津医科大学总医院呼吸内科 Department of Respriatory Medicine, Tianjin Medical University General Hospital, Tianjin 300052, China

## 1 临床资料

患者女性，78岁，退休医生，主因“间断喘息3个月，加重半月”于2011年1月31日入院。患者于入院前3个月无明显诱因出现活动后喘息，无发热、咳嗽、咳痰，无胸痛和心悸，查心电图示“窦性心动过速”，予“倍他乐克”口服，喘息无明显缓解；半月前喘息加重，伴轻咳少痰，查电解质、心肌酶、血常规、脑钠肽等均正常，“倍他乐克”加量无效；入院前1天查胸部CT：左上叶后段可见一肿块影，左侧胸腔积液（图 1）。为求进一步诊治入院，患者自发病以来体重减轻约3 kg。既往多囊肝病史3年；否认结核及外伤、手术史；无吸烟、饮酒史；其父死于“肺心病”，姐姐死于“结肠癌”。

**1:**
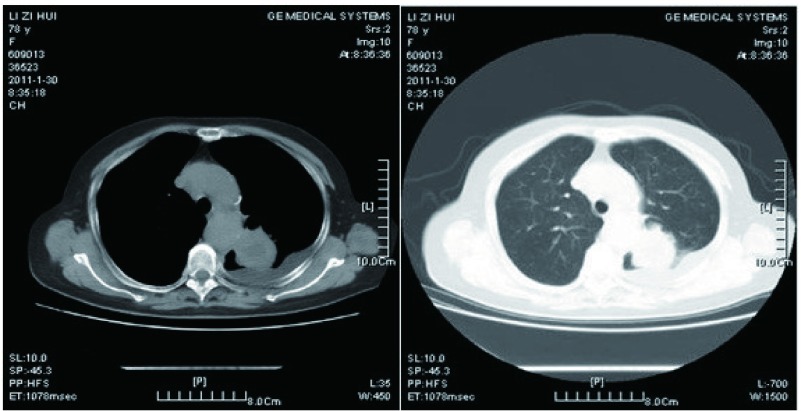
胸部CT示：左上肺肿块，左侧胸腔积液 The chest CT scan revealed a mass on the left upper lobule of the lung with left pleural effusion

入院查体：营养中等，双锁骨上可及多发肿大淋巴结，最大直径约2 cm，质硬、边界清、无压痛、活动差。胸廓对称，左下肺叩浊，呼吸音低，双肺未闻及干湿性啰音。心律齐，各瓣膜区未闻及杂音。腹软，无压痛，肝脾未及，双下肢无水肿，无杵状指。

入院后诊治经过：血尿便常规、肝肾功能和电解质均正常；癌胚抗原、神经原烯醇化酶和细胞角蛋白19片断略高于正常。血脂：总胆固醇TC 5.16 mmol/L（正常范围3.59 mmol/L-5.17 mmol/L），甘油三酯TG 1.73 mmol/L（正常范围0.57 mmol/L-1.71 mmol/L），高密度脂蛋白HDL 0.96 mmol/L（正常范围0.8 mmol/L-2.2 mmol/L），低密度脂蛋白LDL 3.41 mmol/L（正常范围1.33 mmol/L-3.36 mmol/L）。于入院当天行胸腔穿刺置管引流术，为白色混浊乳糜样胸水（图 2），比重1.038，粘蛋白定性试验3+，有核细胞1.0×109/L，中性粒细胞：32%，淋巴细胞：68%，总蛋白86 g/L，腺苷脱氢酶ADA 8.3 U/L，乳酸脱氢酶LDH 212 U/L，胸水TC 3.52 mmol/L，TG 28.58 mmol/L，革兰氏及抗酸染色均阴性，胸水未见肿瘤细胞。支气管镜检查：左上叶升支尖后段开口狭窄，间嵴增宽，病理显示为粘膜慢性炎症。B超示：双侧腋下和颈部、双侧锁骨上及腹股沟区多发淋巴结肿大。PET-CT检查（图 3）示：左肺上叶软组织肿块影，代谢异常增高，考虑为恶性病变伴颈、胸部多发淋巴结、左侧胸膜及双肺转移。左锁骨上淋巴结活检病理示：转移性低分化腺癌（图 4）；免疫组化示：癌细胞呈CK7、TTF-1弥漫性阳性，34βE12散在少许阳性，P63阴性。综合上述检查结果，考虑为左侧低分化肺腺癌伴发乳糜胸。治疗上充分引流胸腔积液后予生理盐水20 mL+香菇多糖2 mg胸膜腔内注射。家属拒绝化疗，接受吉非替尼单药治疗，1周后患者自觉憋气缓解，食欲改善，乏力状况有所改善而出院。

**2:**
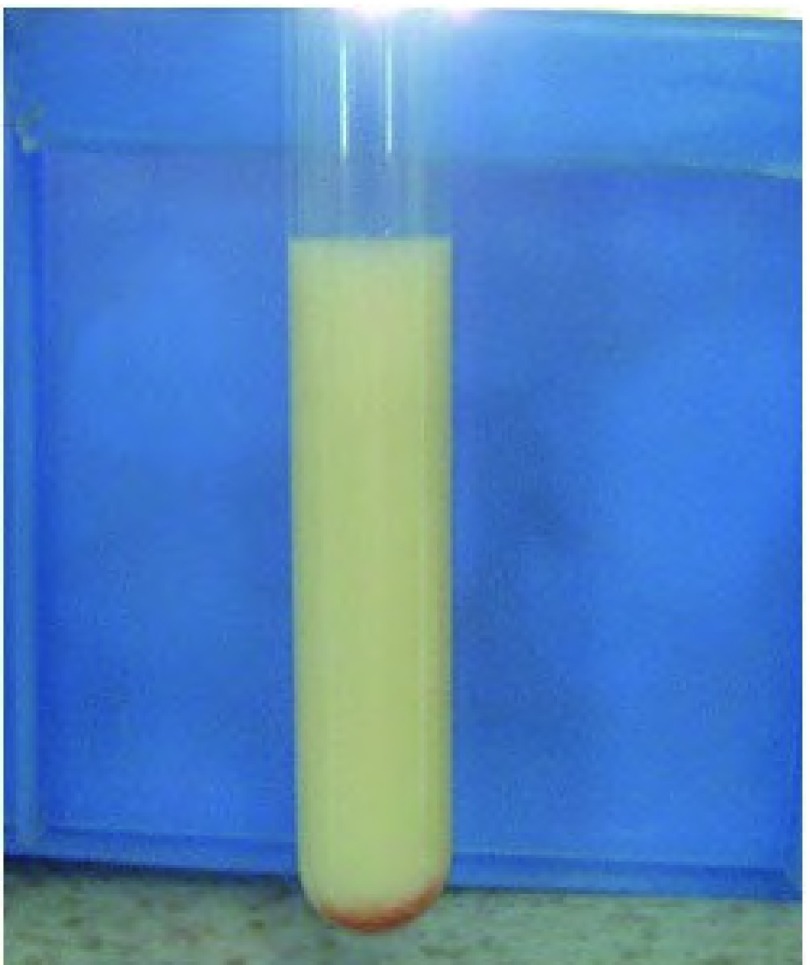
白色乳糜样胸腔积液 White chylous pleural effusion sample

**3:**
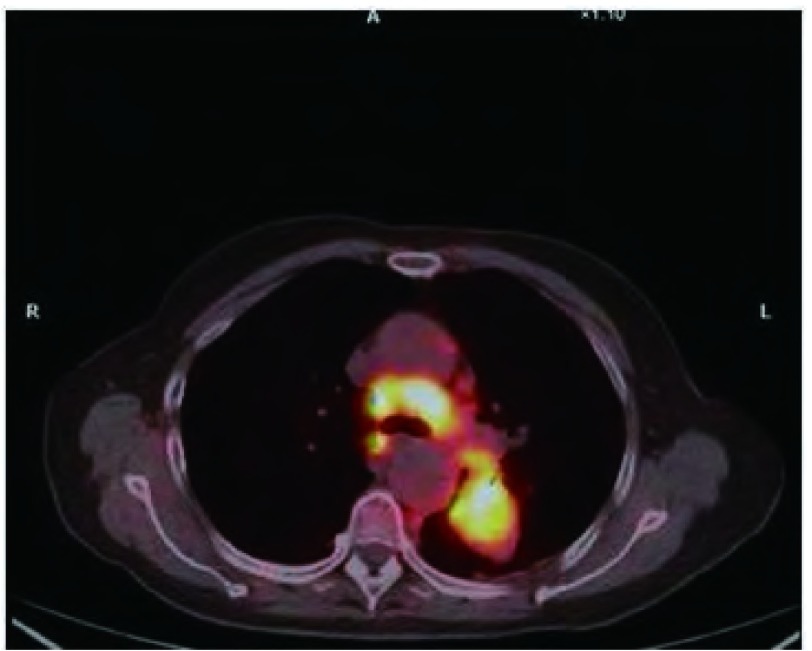
PET-CT示：纵隔内多发大小不等淋巴结，代谢异常增高，左肺上叶软组织肿块影，代谢异常增高 PET-CT scan revealed the enlargement of mediastinal lymph nodes was in multiple sizes, with higher uptake of FDG. The mass on the left upper lobule of the lung also showed higher uptake of FDG

**4:**
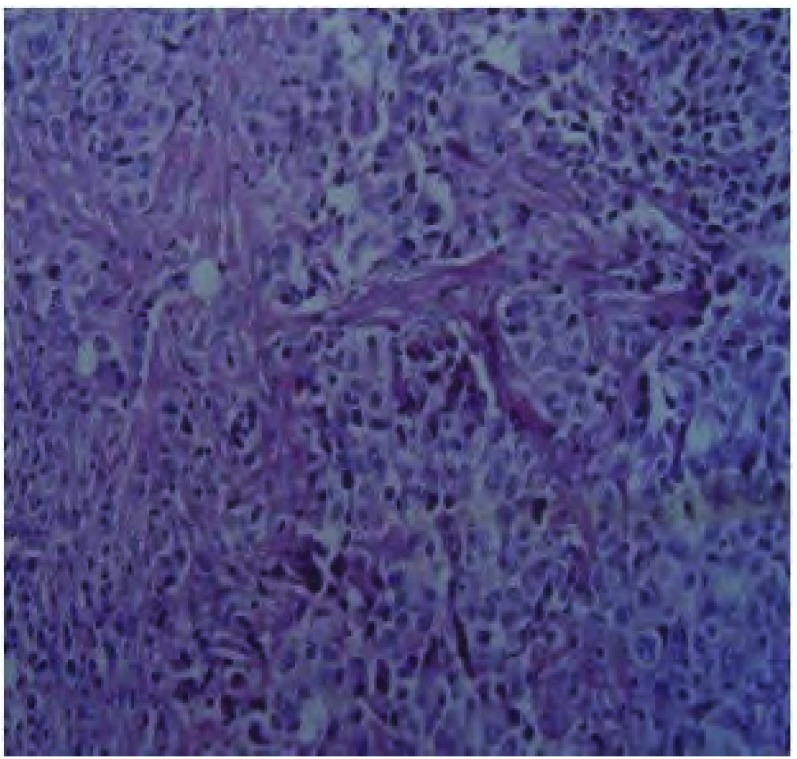
左锁骨上淋巴结活检病理：转移性低分化腺癌（HE, ×100） The left supraclavicular lymph node biopsy demonstrated metastatic poorly differentiated adenocarcinoma (HE, ×100)

## 2 讨论

不同原因导致胸导管或其分支破裂或阻塞，使乳糜液溢入胸膜腔即称乳糜胸（chylothorax）。在临床中，乳糜胸首先需与假性乳糜胸进行鉴别。真性乳糜胸一般呈白色混浊，也可呈浅黄色或粉红色，蛋白>30 g/L，细胞数较少，主要为淋巴细胞（通常>50%），胆固醇含量低，甘油三酯高，胆固醇/甘油三酯 < 1；当胸水甘油三酯>110 mg/dL（1.24 mmol/L）时即有诊断价值，若在胸水中发现乳糜微粒或脂蛋白电泳见到乳糜微粒带则可确诊^[1, 2]^。此外，乙醚实验阳性（即加入乙醚摇荡后因脂肪析出而变清澈），苏丹Ⅲ染色阳性^[3]^。假性乳糜胸是脓胸或胆固醇性胸膜炎所形成的乳状胸水，因积液在胸膜腔内停留时间较长（多>1年），细胞成分坏死、分解和释放胆固醇，使胸液呈乳糜样外观，不含脂肪球及乳糜微粒，胆固醇高达200 mg/dL（5.18 mmol/L），乙醚实验阴性，肉眼或镜下可见折光性强的胆固醇结晶或大量退行性细胞^[4]^。本病例患者胸水为典型的白色混浊乳糜样改变，胸水胆固醇3.52 mmol/L，甘油三酯28.58 mmol/L，胆固醇/甘油三酯 < 1，乳糜胸诊断明确。

乳糜胸约占所有胸腔积液的2%^[3]^，其病因可分为创伤、恶性肿瘤、其它原因及特发性。创伤是引起乳糜胸水最常见的原因，大概占50%^[5]^，其中颈部或胸部的外科手术引起胸导管及其分支的医源性损伤是乳糜胸的主要原因，其它如静脉导管置换术、起搏器植入术、肺动脉栓塞术及胸腔放射等也可引起乳糜胸。恶性疾病是引起乳糜胸的第二大常见原因，约占30%^[5]^，其中由淋巴瘤引起者占到70%-75%，其它恶性肿瘤较为少见^[5]^，而肺癌引起者罕见。台湾台中退伍军人总医院报道的18例恶性肿瘤相关的乳糜胸水病例中，11例患者乳糜胸由淋巴瘤引起，仅1例患者的乳糜胸由肺腺癌引起^[6]^。国内北京协和医院报道的123例乳糜性浆膜腔积液病例中，恶性肿瘤性疾病相关的乳糜性浆膜腔积液病例仅占11例，由淋巴瘤引起者占4例，另外7例分别是胰腺癌、胃癌、食管癌、腹膜间皮瘤各1例，原发部位不详者3例，无肺癌引起的乳糜胸^[7]^。本患者既往否认手术及外伤史，胸CT示左上肺肿物，B超示多发多处淋巴结肿大，临床上需与淋巴瘤进行鉴别，结合PET-CT和左锁骨上淋巴结活检及免疫组化结果考虑为左侧原发性肺腺癌伴发乳糜胸。其它乳糜胸病因包括胸导管栓塞、支气管淋巴结结核、丝虫病、肺淋巴管肌瘤病等。另外，临床中大概有10%不明原因的乳糜胸，称为特发性乳糜胸。

乳糜胸的治疗取决于病因及个体临床状况。患者常因乳糜胸水的丢失引起蛋白质、脂肪及维生素等营养物质的丢失及电解质紊乱，治疗方法包括静脉营养、低脂或中链甘油三酯饮食控制等^[8]^。恶性肿瘤引起者可酌情化疗、放疗或行胸膜固定术。
